# Improved single-cell genome amplification by a high-efficiency phi29 DNA polymerase

**DOI:** 10.3389/fbioe.2023.1233856

**Published:** 2023-06-29

**Authors:** Jia Zhang, Xiaolu Su, Yefei Wang, Xiaohang Wang, Shiqi Zhou, Hui Jia, Xiaoyan Jing, Yanhai Gong, Jichao Wang, Jian Xu

**Affiliations:** ^1^ Single-Cell Center, CAS Key Laboratory of Biofuels, Shandong Key Laboratory of Energy Genetics, Qingdao Institute of Bioenergy and Bioprocess Technology, Chinese Academy of Sciences, Qingdao, Shandong, China; ^2^ Shandong Energy Institute, Qingdao, Shandong, China; ^3^ Qingdao New Energy Shandong Laboratory, Qingdao, Shandong, China; ^4^ University of Chinese Academy of Sciences, Beijing, China; ^5^ CAS Key Laboratory of Biofuels and Shandong Key Laboratory of Synthetic Biology, Qingdao Institute of Bioenergy and Bioprocess Technology, Chinese Academy of Sciences, Qingdao, Shandong, China; ^6^ State Key Laboratory of Microbial Technology, Shandong University, Qingdao, Shandong, China

**Keywords:** single-cell genome amplification, phi29 DNA polymerase, disulfide bond, GB1 fusion protein, process engineering high-efficiency phi29 DNA polymerase

## Abstract

Single-cell genomic whole genome amplification (WGA) is a crucial step in single-cell sequencing, yet its low amplification efficiency, incomplete and uneven genome amplification still hinder the throughput and efficiency of single-cell sequencing workflows. Here we introduce a process called Improved Single-cell Genome Amplification (iSGA), in which the whole single-cell sequencing cycle is completed in a high-efficient and high-coverage manner, through phi29 DNA polymerase engineering and process engineering. By establishing a disulfide bond of F137C-A377C, the amplification ability of the enzyme was improved to that of single-cell. By further protein engineering and process engineering, a supreme enzyme named HotJa Phi29 DNA Polymerase was developed and showed significantly better coverage (99.75%) at a higher temperature (40°C). High single-cell genome amplification ability and high coverage (93.59%) were also achieved for commercial probiotic samples. iSGA is more efficient and robust than the wild-type phi29 DNA polymerase, and it is 2.03-fold more efficient and 10.89-fold cheaper than the commercial Thermo Scientific EquiPhi29 DNA Polymerase. These advantages promise its broad applications in large-scale single-cell sequencing.

## 1 Introduction

Many clinical diagnoses and genetic researches are implemented with cells in bulk, assuming that the cells from the same culture or tissue are identical and functionally synchronous. However, it has been widely recognized that the intercellular variability caused by genetic or exogenous heterogeneity derives from the different microenvironments faced by individual cells in clonal/non clonal populations (Elowitz et al., 2002; Kalisky and Quake, 2011). Therefore, instead of gathering average signals from a heterogeneous population of cells, single cell analysis (SCA) facilitated deciphering single-cell omics and intercellular variations (Haselgruebler et al., 2014). In order to deeply reveal genetic heterogeneity, explore evolutionary mechanisms and uncultured microorganisms, single-cell whole genome amplification (WGA) technology emerged (as reviewed (Kalisky et al., 2011; Blainey, 2013)).

Advances in the rapidly updated next-generation sequencing and whole genome amplification technologies enable high-throughput, multiparameter research on uncultured microorganisms and different aspects of single cell genomes, including single-nucleotide variations (SNVs) and structural variations leading to copy number variations (CNVs) (Navin et al., 2011; Wang et al., 2014). Different DNA amplification techniques have been developed since the middle 1980s, including degenerate oligonucleotide-primed polymerase chain reaction (DOP-PCR), multiple displacement amplification (MDA), and multiple annealing and looping-based amplification cycles (MALBAC) (Gawad et al., 2016). The DOP-PCR and MALBAC methods are more suitable for CNV analysis, because they provide more even genome amplification but lower amplification fidelity (Hou et al., 2015). MDA using the phi29 DNA polymerase, which has special properties of multiple permutation and continuous synthesis, has become the preferred method for investigating SNVs in single cells, as it provides higher genome coverage and fidelity (Wang et al., 2022). Phi29 DNA polymerase, which derives from *Bacillus subtilis* bacteriophage phi29, enables isothermal amplification of trace amount DNA from both circular plasmids and genomic DNAs. It has the capability of incorporating 70,000 nucleotides per event with the fastest rate of DNA synthesis (50–200 bases/s) (Blanco et al., 1989), in addition to proofreading ability that provides higher fidelity, less allele bias, higher sensitivity and efficiency than PCR based methods (Hutchison and Venter, 2006; Lasken, 2007; Hou et al., 2015). Therefore, MDA has been widely applied (Hosono et al., 2003).

However, single-cell MDA suffers from many significant limitations, such as: (a) given the trace amount of DNA from a single cell, substantial contamination DNA from the environment and the generation of primer dimers could largely bias the amplification; (b) preferential amplification of specific genomic regions; (c) generation of chimeric DNA (Yilmaz and Singh, 2012; Huang et al., 2015; Gawad et al., 2016). Several studies improved the performance of MDA focusing on aspects of optimizing the reaction conditions or enzyme engineering (Wu et al., 2006; Marcy et al., 2007; de Vega et al., 2010). For enzyme engineering, based on crystallographic and biochemical studies, the features of phi29 DNA polymerase were modified. The loop of the TP Regions (TPR1) subdomain of phi29 DNA polymerase was studied by changing several residues into alanine, revealing primer-terminus stabilization at the polymerization active site (del Prado et al., 2019). A chimeric phi29 DNA polymerase with helix-hairpin-helix motifs was created in order to enhance salt tolerance and replication performances (Gao et al., 2021). DNA binding ability was improved by the fusion of a Helix-hairpin-Helix domain without affecting the replication rate (de Vega et al., 2010). Furthermore, several polymerase mutants were produced by adding site-directed mutagenesis into the exonuclease active site and C-terminal-conserved regions, which enhanced the incorporation of ribonucleotides and unnatural nucleotides (Blasco et al., 1992; Bonnin et al., 1999; Torres and Pinheiro, 2018). On the other hand, evolutionary methods are used for DNA polymerase engineering through the method of compartmentalized self-replication (CSR) (Ghadessy et al., 2001; Ghadessy and Holliger, 2007; Abil and Ellington, 2018; Sakatani et al., 2019). For reaction conditions optimization, host DNA and environmental DNA may lead to false positive results in the amplification reaction, as phi29 DNA polymerase has high affinity for both single-strand and double-stranded DNA (Blanco et al., 1989). DNA contaminants in the reagents affect amplification too. Ethidium monoazide, ultraviolet-free light-emitting diode lamp and trehalose can remove contaminants from host DNA, laboratory environments, tools and reagents (Takahashi et al., 2014). Smaller WGA volume can increase the concentration of the template and lessen the chance of background contamination amplification (Sobol and Kaster, 2023). There are several microbial single-cell genomics volume reduction methods including microfluidic devices (Ruan et al., 2020), nanowells (Goldstein et al., 2017), planar surfaces (Rezaei et al., 2021), and hydrogels (Xu et al., 2016), which significantly reduce amplification bias and increase assembly coverage up to almost 90% (Sobol and Kaster, 2023). Moreover, since elevated reaction temperature reduced the influence on amplification bias, MDA reaction was reported to perform better when amplification temperature increased (Henry and Romesberg, 2005). Therefore, in order to apply Phi29 DNA polymerase to WGA and single cell genomics amplification, a systematic engineering, including enzyme engineering and process engineering is required.

**FIGURE 1:**
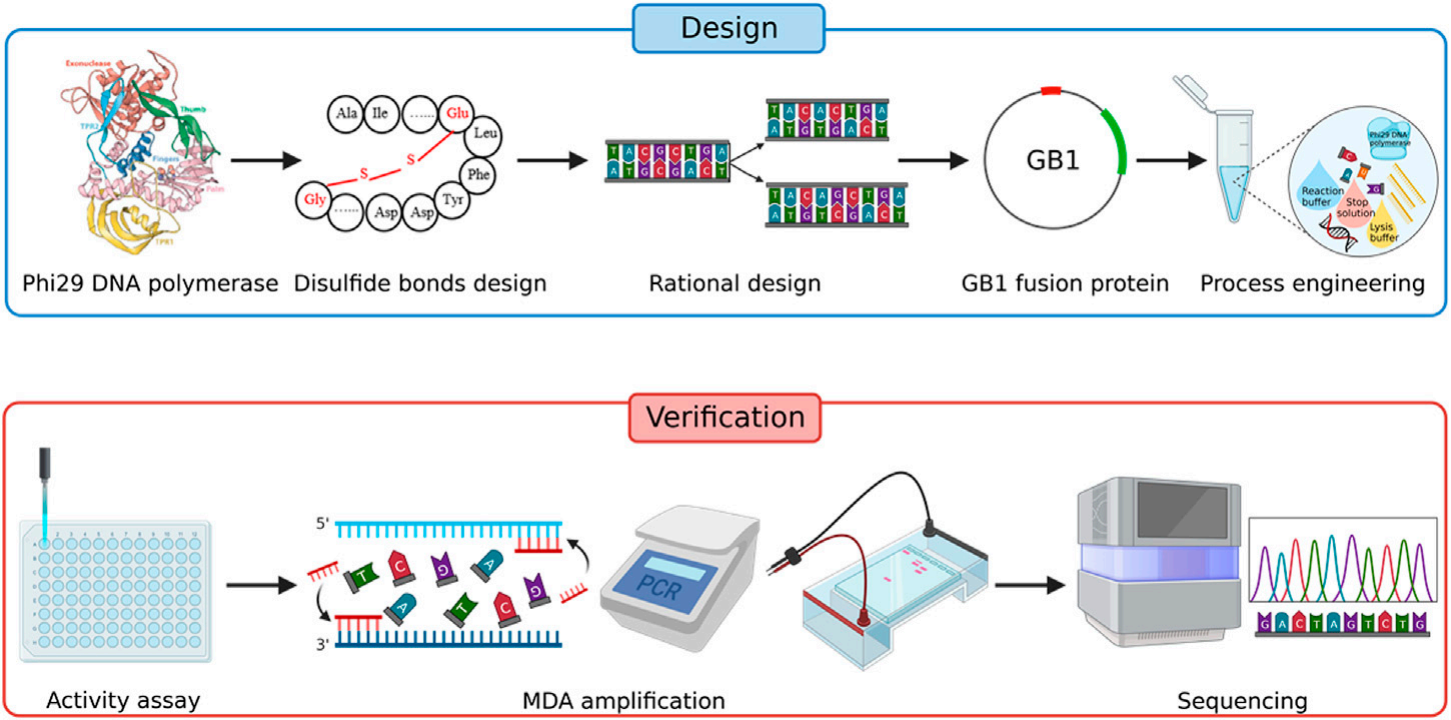
Strategy for improving the amplification efficiency of phi29 DNA polymerase.

Here we present an improved single-cell genome amplification method called Improved Single-cell Genome Amplification (iSGA) by phi29 DNA polymerase modification and enzyme reaction system optimization (Figure 1). This is achieved by the combination of rational protein engineering and process engineering that extended its micro template amplification ability and improved the coverage of single-cell genome amplification. As a result, a supreme enzyme named HotJa Phi29 DNA Polymerase was developed and shows significantly better coverage (99.75%) at a higher temperature (40°C). For commercial probiotic samples, HotJa Phi29 DNA Polymerase still showed high single-cell genome amplification ability and high coverage (93.59%). iSGA is more efficient and robust than the wild-type phi29 DNA polymerase, and it is 2.03-fold more efficient and 10.89-fold cheaper than Thermo Scientific EquiPhi29 DNA Polymerase which is the most effective enzyme that has been reported. These advantages promise its broad applications in industrial single-cell sequencing.

## 2 Materials and methods

### 2.1 Reagents and microorganisms

All chemicals used were of analytical grade or higher. LB (Luria-Bertani) media, IPTG (isopropyl-β-d-thiogalactoside), and ampicillin were obtained from Sangon Biotech (Shanghai, China). T4 DNA ligase and all restriction enzymes were obtained from Thermo Fisher Scientific Inc. (MA, United States). KOD Plus DNA polymerase was from TOYOBO (Osaka, Japan), Q5 High-Fidelity DNA polymerase was from New England Biolabs (NEB, MA, Ipswich, United States). *Escherichia coli* BL21 (DE3) and DH5α and plasmid pET-21c (+) were obtained from Sangon Biotech (Shanghai, China). LB medium was used to aerobically culture all the strains. To prepare solid plates of each medium, 15 g/L agar was added. *E. coli* DH5α was used for cloning. *E. coli* BL21 (DE3) was used for expressing the phi29 proteins. Plasmid pET-21c (+) was used for GB1-phi29 fusion protein expression. All purification buffers and reaction buffers were prepared, containing magnesium chloride (MgCl_2_), potassium chloride (KCl), ammonium sulfate (NH_4_)_2_SO_4_, tris hydrochloride, dithiothreitol (DTT), tween-20, Nonidet P-40, ethylenediaminetetraacetic acid disodium salt (EDTA), imidazole, glycerol, potassium hydroxide (KOH) that were purchased from Sangon Biotech (Shanghai, China). The concentrations of HotJa Phi29 DNA Polymerase were measured by Bradford Protein Assay Kit (Sangon Biotech, Shanghai, China). The enzyme activity was measured, using SYBR green I (Beijing Solarbio Science & Technology Co., Ltd.), random hexamer primer (6N; Integrated DNA Technologies, IDT, Coralville, United States), deoxy-ribonucleoside triphosphate (dNTPs; Vazyme, Nanjing, China), HindIII-digested λ DNA (New England Biolabs, NEB, Ipswich, MA, United States). All primers used in this study are summarized in Supplementary Table S1. All plasmids and strains constructed are described in Supplementary Materials and summarized in Supplementary Table S2 and Supplementary Table S3.

### 2.2 Design of disulfide bonds

According to the X-ray crystallography structure of phi29 DNA polymerase derived from *Bacillus phage* (PDB: 1XI1), the disulfide bonds were introduced using the software Disulfide by Design (DbD, version 2.12) (Craig and Dombkowski, 2013). The top 12 disulfide bonds supposed to stabilize the protein most significantly were subjected to experimental validation. All disulfide bonds constructed in this study are listed in Table 1.

**TABLE 1:** The disulfide bonds constructed in this study.

Plasmid	Amino acid mutation	Gene mutation
pZJphi29001	K7C-A56C	AAA21TGC—GCG168TGC
pZJphi29002	V24C-L44C	GTT72TGC—CTG132TGC
pZJphi29003	Y59C-S122C	TAC177TGC—AGC366TGC
pZJphi29004	E75C-L406C	GAA225TGC—CTG1218TGC
pZJphi29005	F137C-A377C	TTT411TGC—GCG1131TGC
pZJphi29006	S194C-S388C	TCC582TGC—TCT1164TGC
pZJphi29007	L253C-D458C	CTG759TGC—GAC1374TGC
pZJphi29008	Y254C-Y390C	TAC762TGC—TAC1170TGC
pZJphi29009	M258C-F360C	ATG774TGC—TTC1080TGC
pZJphi29010	Y281C-K352C	TAC843TGC—AAA1056TGC
pZJphi29011	Y298C-H339C	TAC894TGC—CAC1017TGC
pZJphi29012	V331C-A435C	GTG993TGC—GCC1305TGC

### 2.3 Expression and purification of phi29 DNA polymerase

The wild-type and mutants of phi29 DNA polymerase that contains double or multiple amino acid mutations were purified through Ni-NTA immobilized metal affinity chromatography (IMAC) as follows: 2 mL of the *E. coli* BL21 (DE3) cells harboring the corresponding polymerase over-night culture were added to 200 mL LB medium containing 100 mg/L ampicillin and shaken at 37°C. When the OD600 reached approximately 0.9 that measured by ultraviolet spectrophotometer (NP80, Implen GmbH, Munich, Germany), IPTG was added at the final concentration of 1 mM to induce protein expression with additional shaking at 16°C for 21 h. The culture was harvested by centrifugation for 5 min at 4°C and 7000 g (3-18KS, Eppendorf, Hamburg, Germany) and re-suspended in lysis buffer (20 mM Tris-HCl (pH 7.6), 300 mM KCl, 1 mM EDTA, 5 mM imidazole). After sonication (JY92-IIDN, Scientz, Ningbo, China) on ice for 45 min, samples were collected through centrifugation for 20 min at 4°C and 15000 g. The supernatant was loaded onto a ÄKTA pure (Cytiva, United States) with Ni-NTA affinity column (HISTRAP HP, Cytiva, United States) previously equilibrated with lysis buffer. In order to remove the un-specifically bound proteins, the column was washed with wash buffer I (20 mM Tris-HCl (pH 7.6), 300 mM KCl and 40 mM imidazole) and wash buffer II (20 mM Tris-HCl (pH 7.6), 300 mM KCl and 80 mM imidazole). The his-tagged proteins were eluted using elution buffer (20 mM Tris-HCl (pH 7.6), 300 mM KCl and 250 mM imidazole). Eluted polymerases were dialyzed against storage buffer (20 mM Tris-HC1(pH7.6), 100mM KC1, 0.5% (v/v) Tween 20, 0.1 mM EDTA, 1 mM DTT, 50% glycerol) and stored at −20°C.

### 2.4 The measurement of Phi29 DNA polymerase activities

The amount of phi29 polymerase that incorporate 0.5 pmol of dNTP into acid insoluble material in 10 min at 30°C was defined as one unit. Firstly, a standard curve for the relationship between fluorescence intensity and enzyme activity units was established using a commercial reagent kit (EquiPhi29™ DNA Polymerase, Thermo Fisher Scientific Inc., MA, United States). 20 μL reaction systems containing 1× phi29 DNA polymerase reaction buffer, 0.01 mg/mL Hind II-digested λ DNA (New England Biolabs, NEB, Ipswich, MA, USA) and 0.5 μM random hexamer primer (6N; Integrated DNA Technologies, IDT, Coralville, USA) were incubated at 95°C for 3 min and placed on ice. Then final concentrations of 0.2 mM dNTPs (Vazyme, Nanjing, China), 0.1 mg/mL BSA (Sangon Biotech Co., Ltd., Shanghai, China), diluted EquiPhi29 DNA polymerase, and 1× SYBR green I (Beijing Solarbio Science & Technology Co., Ltd.) were added to a final volume of 50 μL. The dilution ratios of EquiPhi29 DNA polymerase were 2, 4, 6, 8, 10 times, and each ratio had three replications. Secondly, the reaction systems were incubated at 30°C for 4 h and 65°C for 10 min for inactivation. Finally, microplate reader was used to excite at 480 nm and measure the absorption at 520 nm for fluorescence intensity to establish the standard curve. HotJa Phi29 DNA Polymerase was used for the same reaction above, and the activity was calculated according to the standard curve.

### 2.5 Process engineering for improving iSGA

The optimization of iSGA contains phi29 DNA polymerase modification and reaction process optimization. To optimize the stability and enzymatic activity of phi29 DNA polymerase, we evaluated the effects of salt concentrations and several components. In order to determine the relationship between KCl concentrations and enzymatic activity, the phi29 DNA polymerase was dialyzed to 50 mM, 100 mM, 150 mM, 200 mM, and 300 mM KCl, respectively. Furthermore, several components were added to the storage buffer such as EDTA, DTT, tween 20 and NP-40 so as to improve the stability of phi29 DNA polymerase. What is more, UV-irradiation has been implemented to suppress the amplification of unwanted DNA. We had evaluated the effect of UV exposure time to the MDA reaction reagents on removing contaminant DNA as well as the enzymatic activity through a time-course assay. The phi29 DNA polymerase and MDA reaction reagents were exposed to UV-irradiation for 0, 5, 10, 30, 60 and 120 min before the MDA reaction and 16S rRNA amplification were conducted to measure the DNA contamination elimination efficiency of different UV irradiation durations.

### 2.6 Single cell isolation through easysort platform

The single bacterial cell was indexed and sorted through Easysort platform (Single-Cell Biotech., Qingdao, China) with microfluidics-chip (SCB-D002, Single-Cell Biotech., Qingdao, China) which was an upgrade from Raman-Activated Gravity-driven single-cell Encapsulation (RAGE) platform (Xu et al., 2020). Briefly, the Easysort platform utilized the gravity force generated through the height-adjustable sample holder to achieve precise flow-control of the cells. In addition, a mathematical mode was built to investigate the key factors that influence droplet dimension and guide the chip design, at the end, reliable single-cell encapsulation, generation of droplets with small sizes (picoliter level) and good uniformity was achieved. In combination, Easysort platform can achieve single cell isolation in a precise, high-throughput and indexed manner.

### 2.7 Assessing the method of iSGA through MDA

Single bacterial cells were sorted by Easysort (Single-Cell Biotech., Qingdao, China) with microfluidics-chip (SCB-D002, Single-Cell Biotech., Qingdao, China). 30-mm-width and 10-mm-depth microchannel was used for the sorting of microbial cells. Sorted cells in the microtubes were lysed with 1 μL lysis buffer (A buffer; Single-Cell Biotech., Qingdao, China) at 65°C for 10 min, followed by addition of 1 μL stop solution (B buffer; Single-Cell Biotech., Qingdao, China). Then 10× HotJa-reaction buffer (0.4 M Tris-HC1(pH7.6), 0.5 M KCl, 50 mM (NH4)_2_SO_4_, 100 mM MgCl_2_, 40 mM DTT) and HotJa Phi29 DNA Polymerase [for control, amplified with EquiPhi29 DNA polymerase and buffer (Thermo Fisher Scientific, United States)] were added to each tube, and thoroughly mixed and incubated at 40°C for 8 h with 70°C heated lid on a thermocycler (T100, Bio-Rad, California, United States). Reactions without cells or DNA template were carried out as negative control in parallel to quantify potential contamination. The amplified DNA was verified on 1% agarose gel and served as PCR template for amplification of 16S-rRNA (via the 27F and 1492R primers; Supplementary Table S1) (Edwards et al., 1989; Weisburg et al., 1991) and *uspA* gene (via the uspA-F and uspA-R primers; Supplementary Table S1) (Chen and Griffiths, 1998) after 20-fold dilution.

## 3 Results

### 3.1 Wild-type phi29 DNA polymerase suffers from low amplification efficiency

The wide-type phi29 DNA polymerase’s amplification efficiency is too low to complete droplet-based single-cell level genome amplification. Specifically, in the *E. coli* single-cell level genome amplification experiment, wide-type phi29 DNA polymerase could complete MDA reaction with extracted genomic DNA (fg level) as template, but it could not complete with single-cell as template (Supplementary Figure S1). One research has reported that the MDA reaction was successfully carried out at 40°C only in the case of mutant polymerase and completely failed with wide-type polymerase, which generated a significantly higher amount of MDA products. The amplification efficiency of the mutant polymerase at 40°C (∼30,000-fold) was about six times higher than that of the wild-type enzyme at 30°C (∼6,000-fold) (Povilaitis et al., 2016). Consequently, the wide-type phi29 DNA polymerase could only complete MDA reaction with trace extracted genomic DNA as template, which is not suitable for single-cell analysis.

### 3.2 Introduction of specific disulfide bonds improves phi29 DNA polymerase amplification efficiency

**FIGURE 2:**
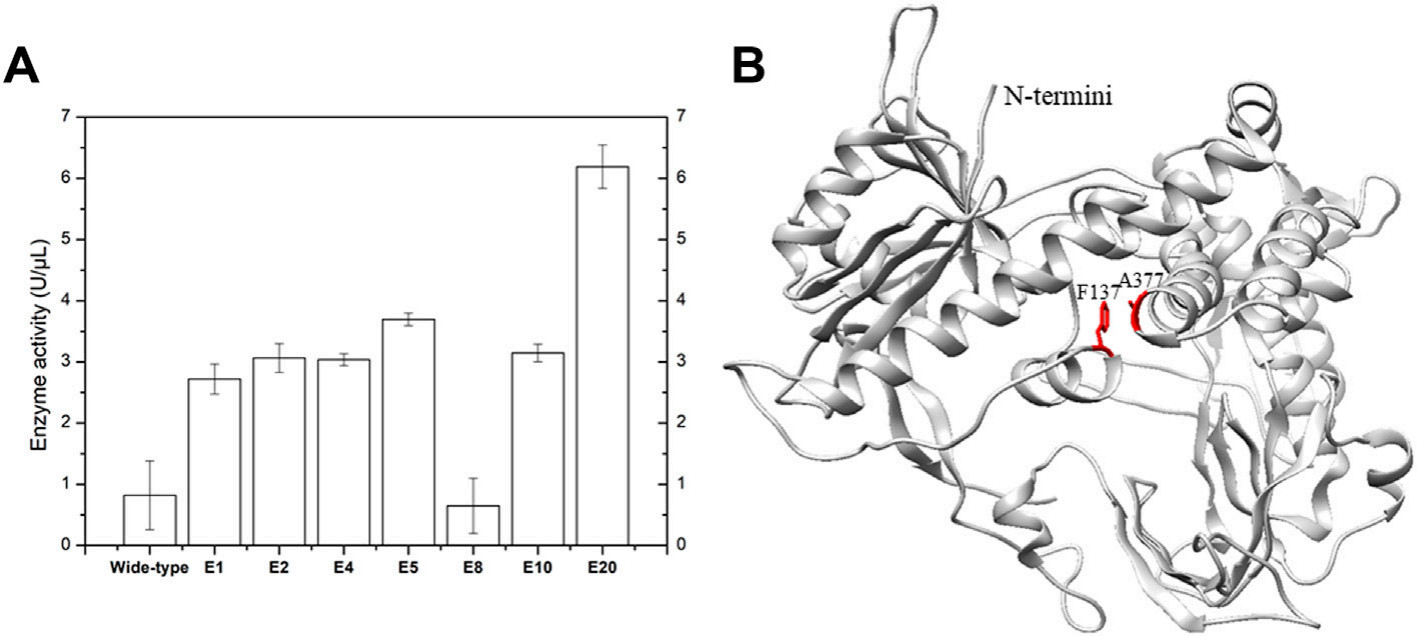
Specific disulfide bonds and point mutations improve phi29 DNA polymerase amplification efficiency. **(A)** Elevation of phi29 enzyme activity by introducing specific disulfide bonds. E1, E2, E4, E5, E8, E10 and E20 represent the results of EZJphi29001, EZJphi29002, EZJphi29004, EZJphi29008, EZJphi29010 and EZJphi29020. **(B)** The structure of phi29 DNA polymerase. The structure of phi29 DNA polymerase (PDB: 1XI1) was shown in grey cartoon, F137 and A377 were shown in red sticks.

**FIGURE 3:**
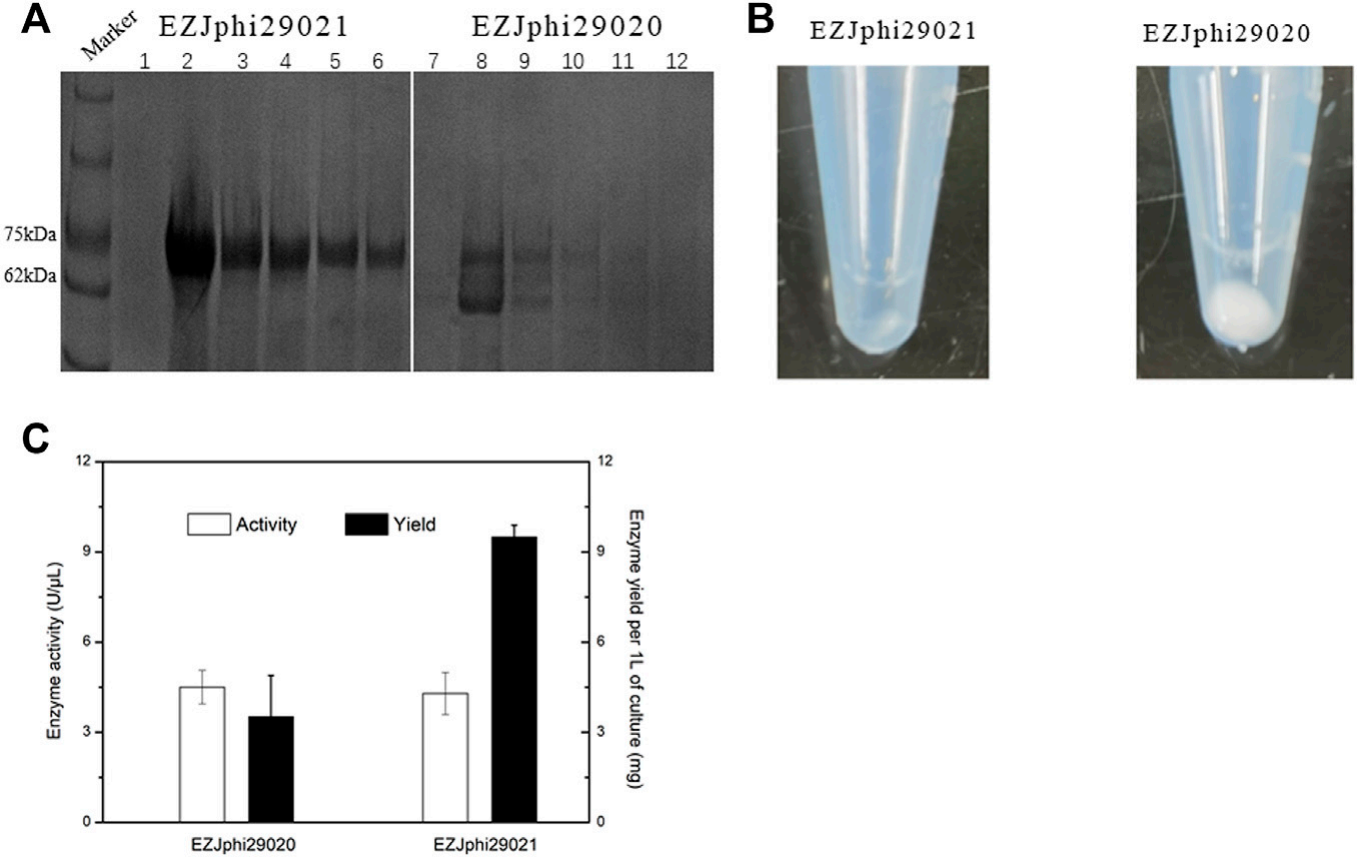
Fusion to GB1 improved the expression level of Phi29. **(A)** 8% sodium dodecyl sulfate-polyacrylamide gel electrophoresis (SDS-PAGE) analysis of the expression and solubility of two versions of mutant phi29 DNA polymerase. Lane 1–6 for EZJphi29021, and lane 7–12 for EZJphi29020. **(B)** Precipitation photos demonstrating the solubility of two versions of mutant phi29 DNA polymerase. Left for EZJphi29021, and right for EZJphi29020. **(C)** Comparison diagram of the enzymatic activity and enzyme yield of two versions of mutant phi29 DNA polymerase.

It has been reported that performing MDA at elevated temperature can drastically improve the specificity, amplification yield and limit templet-independent DNA amplification (TIDA) (Alsmadi et al., 2009). The introduction of disulfide bonds through molecular modeling could drastically increase the chemical, pH and thermal stability of proteins (Perry and Wetzel, 1984; Matsumura et al., 1989; Liu et al., 2019). To improve its stability, phi29 DNA polymerase was engineered by introducing specific disulfide bonds (Figure 2; Table 1). Among all the constructed disulfide bonds phi29 DNA polymerase mutants, EZJphi29003, EZJphi29006, EZJphi29007, EZJphi29009, EZJphi29011, EZJphi29012 had lost enzymatic activity; EZJphi29001, EZJphi29002, EZJphi29004, EZJphi29008, EZJphi29010 could only complete MDA reaction with trace extracted genomic DNA but not with single cell as template, while EZJphi29005 succeeded in conducting MDA reaction with both trace extracted genomic DNA and single cell as template, the rate of 16S rRNA amplification was 100%. This result indicates that introducing disulfide bonds at positions F137 and A377 could stabilize and improve the amplification ability of phi29 DNA polymerase compares to the wild type (Figure 3; Supplementary Figure S1). Introduction of disulfide bonds at positions Y59-S122, S194-S388, L253-D458, M258-F360, Y298-H339, V331-A435 could impair the enzymatic activity, and at positions K7-A56, V24-L44, E75-L406, Y254-Y390, Y281-K352 have no apparent effect on the enzymatic activity of phi29 DNA polymerase (Figure 3; Supplementary Figure S1). Finally, phi29-F137C-A377C was chosen for subsequent single cell amplification experiments.

### 3.3 Introduction of mutants further improves phi29 DNA polymerase amplification efficiency

By implementing modified compartmentalized self-replication method (iCSR), phi29 DNA polymerase mutants (M8R, V51A, M97T, G197D, E221K, Q497P, K512E, F526L) were identified, which can complete MDA at elevated temperature (42°C), produce 2–5 times more products, and have significantly increased half-life (Povilaitis et al., 2016). According to the experimental results and literature review (Loh and Loeb, 2005), those eight mutations have several positive effects associated with the amplification efficiency of mutant phi29 DNA polymerase, including stability effect, protein affinity to the DNA substrate and so on. In this case, the M8R and M97T mutation sites may increase the protein affinity to DNA substrate in order to increase amplification efficiency. The K512E mutation site increases residence time for nucleotide analogue incorporation that improves processivity, retention time, surface stability and affinity tagging. The F526L mutation site improves the polymerase resistance to photodamage. The Q497P mutation site has an effect on the stabilization of the polymerase-substrate complex and DNA synthesis fidelity. Therefore, we further constructed EZJphi29020 mutant which contains 10 point mutants (F137C, A377C, M8R, V51A, M97T, G197D, E221K, Q497P, K512E, F526L) and could complete MDA reaction with both trace extracted genomic DNA and single cell as template and showed higher thermal stability (Figure 2; Supplementary Figure S1).

### 3.4 Fusion to GB1 protein increases the protein’s expression level

Despite the stabilization effect of introducing disulfide bonds to the phi29 DNA polymerase, the high number of disulfide bonds could also lead to aggregation of folding intermediates and generation of inclusion bodies due to the reductive environment in cytoplasm of *E. coli* cells (de Marco, 2009). Several strategies have been implemented to prevent the aggregation of proteins by shielding hydrophobic patches on their external surfaces that include: supplying detergents, recombinant expression with highly soluble chaperons, co-expressing isomerase DsbC (de Marco, 2009). In this work, the 56-residue GB1 (B1 immunoglobulin binding domain of *Streptococcal* protein G), which has been used as small fusion partner to improve the expression level, solubility and stability of the fusion protein (Gronenborn et al., 1991; Bao et al., 2006), was over-expressed (Figure 2). By fusing GB1 to the N-terminal of phi29 DNA polymerase (named EZJphi29021 or HotJa DNA polymerase), the expression and solubility of two versions of mutant phi29 DNA polymerase were demonstrated by 8% SDS-PAGE analysis (Figure 3A) and precipitation photos (Figure 3B). A 2.7-fold increase of yield was observed in GB1-tagged phi29 DNA polymerase (Figure 3C). The expression and purification of mutant phi29 DNA polymerase in large-scale is a challenge in researches associated with functionality analysis of phi29 DNA polymerase (Sakatani et al., 2019). This result has provided an effective strategy to increase the solubility and maintain the activity of phi29 DNA polymerase at the same time.

### 3.5 Process engineering for improving the method of iSGA

**FIGURE 4:**
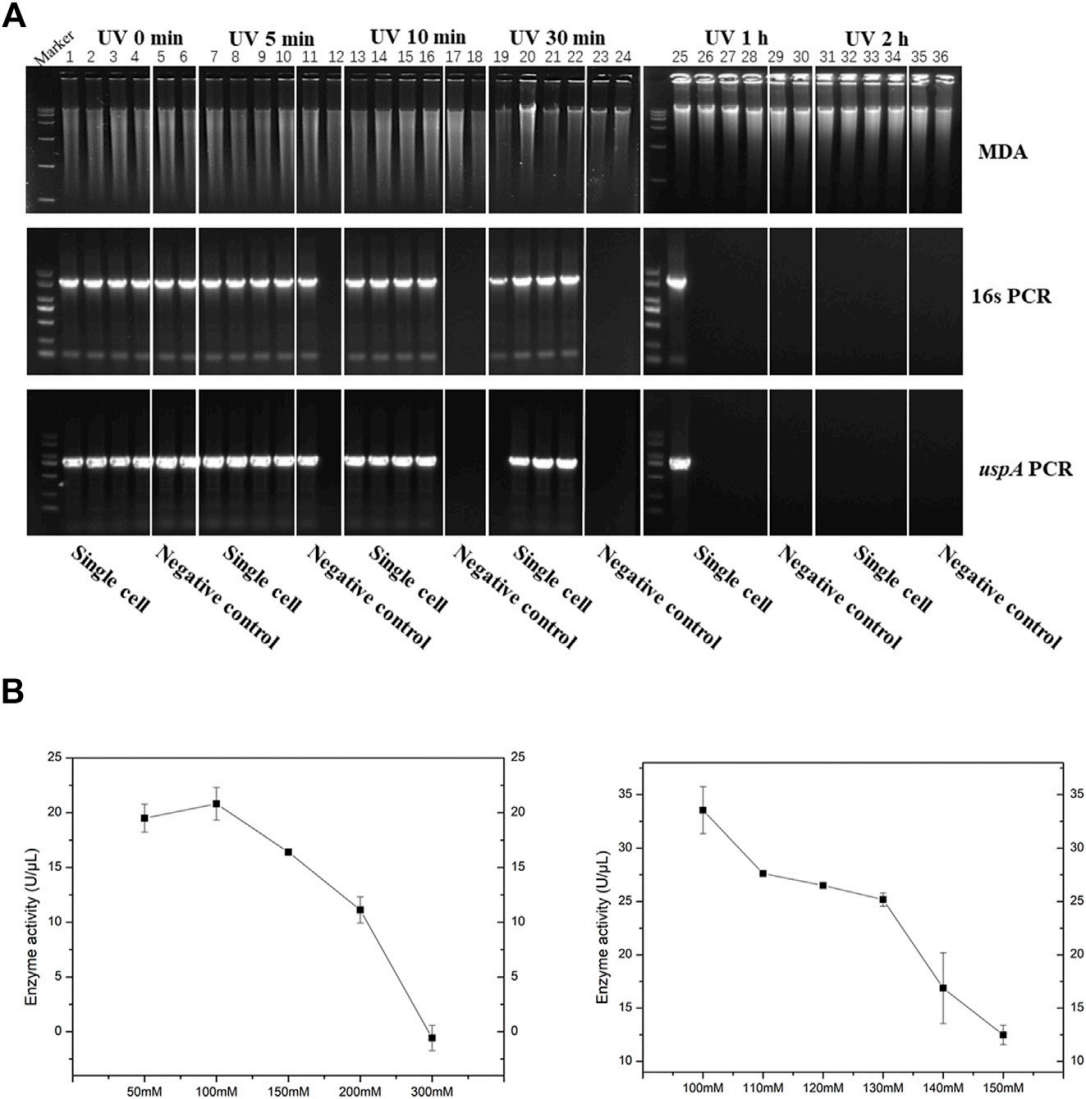
Process engineering improved MDA. **(A)** UV-irradiation treatment improved MDA. 1% agarose gel analysis of the amplification efficiency of phi29 DNA polymerase under different UV irradiation time. Lane 1–6 for UV irradiated 0 min, lane 7–12 for UV irradiated 5 min, lane 13–18 for UV irradiated 10 min, lane 19–24 for UV irradiated 30 min, lane 25–30 for UV irradiated 1h, and lane 31–36 for UV irradiated 2 h. Lane 1–4, 7–10, 13–16, 19–22, 25–28 and 31–34, single cell was amplified by EZJphi29021 that under different UV irradiation time. Lane 5,6,11,12,17,18,23,24,29,30,35,36 for negative control. 16s PCR indicates the PCR results with primers 27F and 1492R, *uspA* PCR indicates the PCR results with primers uspA-F and uspA-R. **(B)** The relationship between the concentration of potassium ion and enzyme activity.

In this work, we presented and discussed several results about two aspects of the process engineering phase for improving the method of iSGA, including efficient decontamination method and buffer modification. Firstly, we evaluated the effect of suitable UV-irradiation treatment of MDA reagents that eliminates the contaminating DNA and maintains the activity of high purity phi29 DNA polymerase at the same time. The MDA reaction mixtures and HotJa Phi29 DNA Polymerase were irradiated for 0, 5, 10, 30, 60, 120 min prior to MDA of single *E. coli* cell (Figure 4A). We observed a decreasing success rate of amplified single cells with an increase of UV treatment times. With UV-irradiation treatment at 10 min, the success rate of amplified single cells was 100% (0% of negative control), which revealed the contaminating DNA was effectively eliminated under 10 min UV irradiation, as well as the enzymatic activity was not affected. In contrast, if the UV treatment time was shorter or longer than 10 min, the contaminated DNA was not removed clearly or the enzymatic activity decreased, respectively (Figure 4A). There are several studies and application claims that have presented a key challenge inherent to MDA reaction is DNA contamination (Lee, 2011; Woyke et al., 2011). While DNA-free reagents are a prerequisite for single cell amplification in order to successfully conduct single cell sequencing and analysis (Lee, 2011). Those studies have comprehensively demonstrated the utility of UV irradiation to effectively eliminate exogenous contaminant DNA found in MDA reagents with higher enzymatic activity at the same time (Lee, 2011). Meanwhile, genome coverage of MDA amplified single cell is not adversely effected during the increase of UV expose times (Woyke et al., 2011) (Table 2).

**TABLE 2:** Precise one-cell genome sequencing of E.coli via iSGA compared with Equiphi29 DNA polymerase.

	Sample	Coverage (%)
HotJa	E3	89.39
	E1	99.75
	E5	99.75
	E26	97.47
	E18	95.59
	E16	90.89
	E7	92.21
	E22	99.7
	E21	88.87
Equiphi29	E17	33.42
	E2	27.65
	E6	20.86
	E14	82.84
	E24	73.83
	E32	33.45
	E34	18.78
	E38	52.4
	E45	76.29

On the other hand, we analyzed and assessed the component contents in MDA reaction buffer and phi29 DNA polymerase storage buffer that increase the amplification efficiency and reduce enzymatic degradation (precipitation, e.g., proteolysis), simultaneously. We evaluated the relationship between the concentration of potassium ions with 50, 100, 150, 200, 300 mM and enzymatic activity (Figure 4B). As a result, when the concentration of potassium ions was 100 mM, the phi29 DNA polymerase activity was highest at about 21 U/μL. Meanwhile, the phi29 DNA polymerase will begin to precipitate when the concentration of potassium ion was 50 mM (Figure 4B). In addition, some additives like non-ionic detergents (e.g., Triton X-100 and Tween 20) were used in this study to stabilize the activity of DNA polymerase (Wu and Cetta, 1975).

### 3.6 iSGA is efficient and cost-effective in the WGA workflows

**FIGURE 5:**
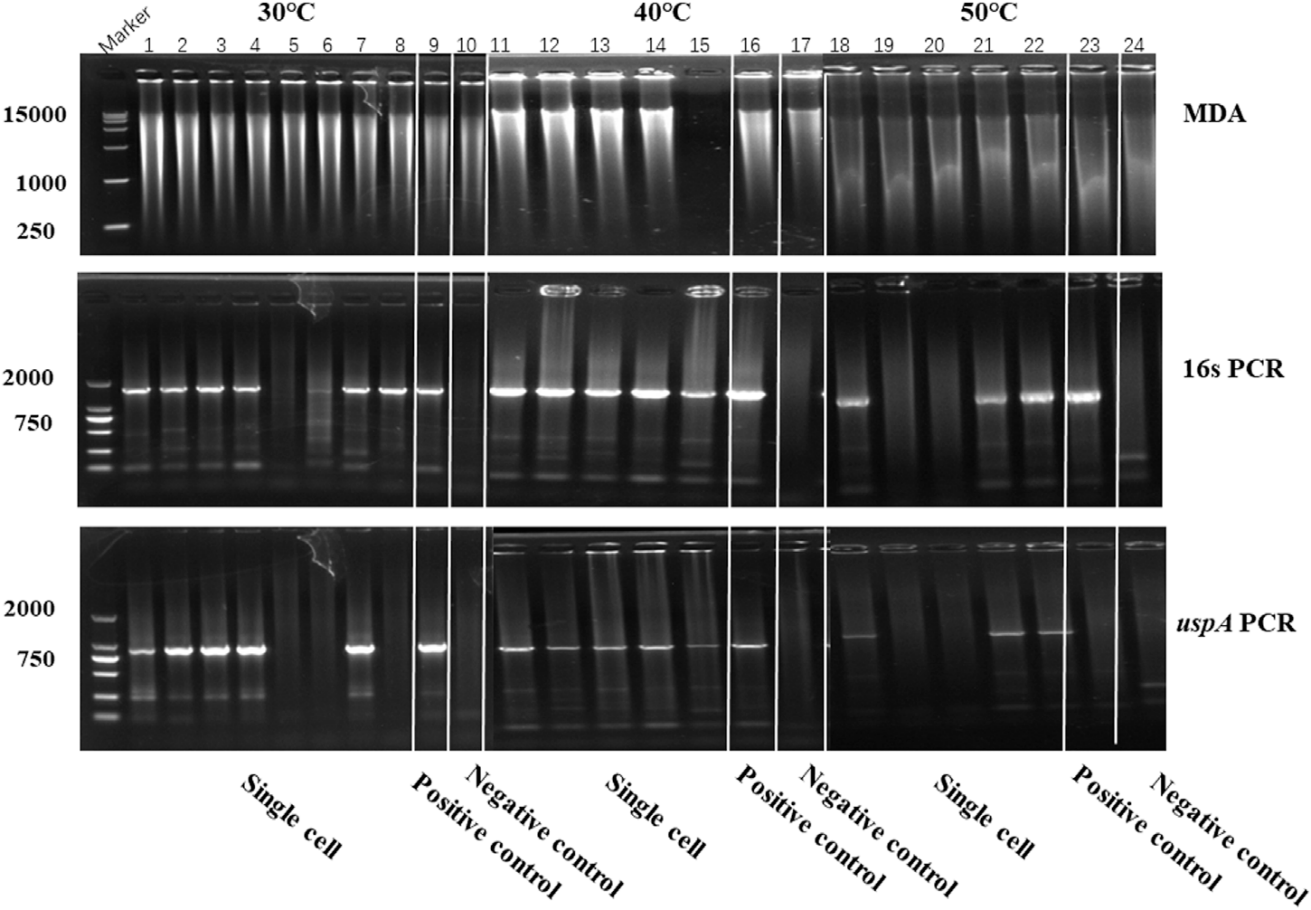
The amplification efficiency of HotJa Phi29 DNA polymerase at different reaction temperatures. Lane 1–10 for MDA reactions performed at 30°C, lane 11–17 for MDA reactions performed at 40°C, lane 18–24 for MDA reactions performed at 50°C. Lane 1–8, 11–15 and 18–22, single cell was amplified by HotJa Phi29 DNA polymerase at 30°C, 40°C, 50°C, respectively. Lane 9, 16 and 23 for positive control. Lane 10, 17 and 24 for negative control. 16s PCR indicates the PCR results with primers 27F and 1492R, *uspA* PCR indicates the PCR results with primers uspA-F and uspA-R.

**FIGURE 6:**
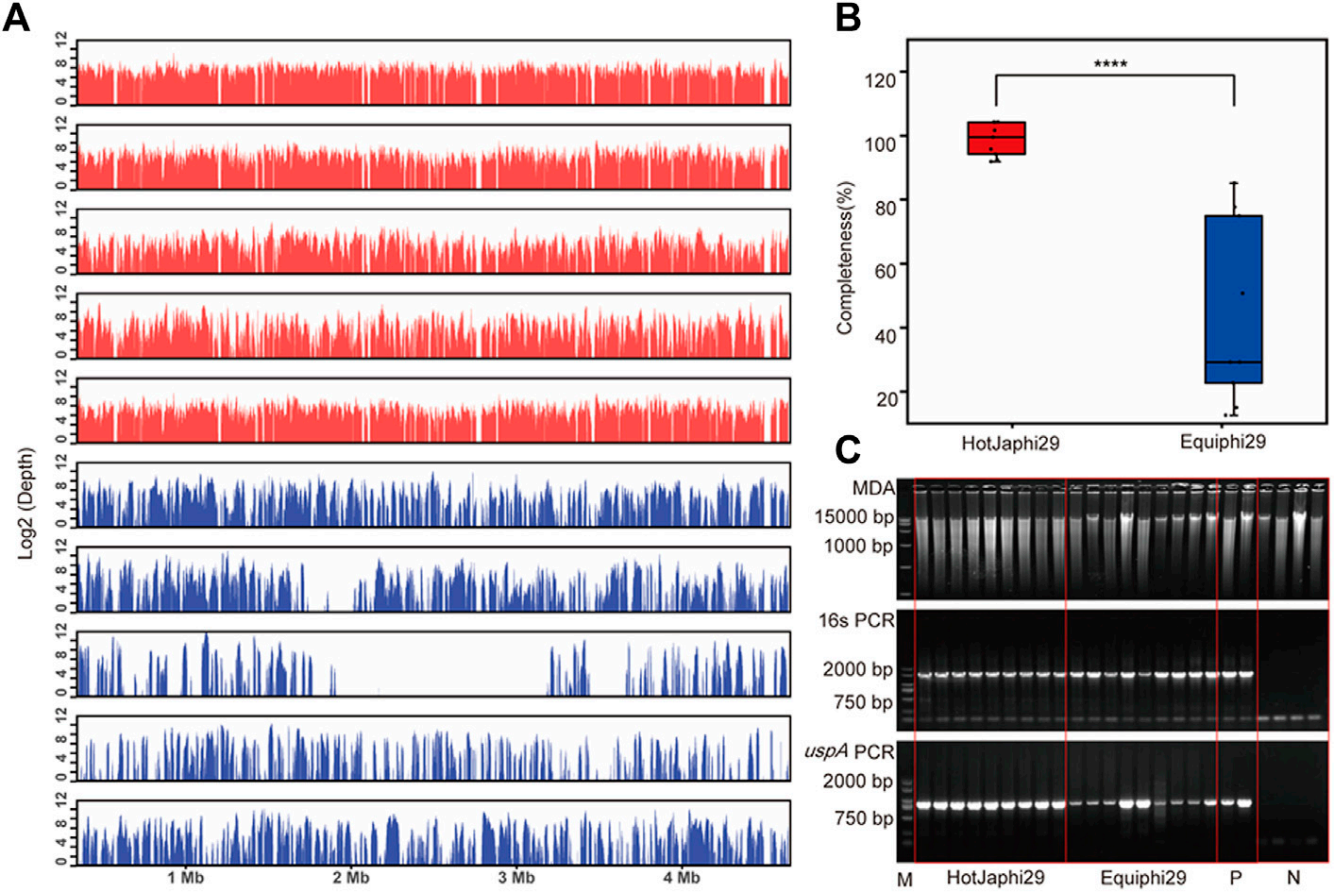
Comparison of HotJa and Equiphi29 DNA polymerase in (E) *coli* single cell amplification. **(A)** Read coverage distribution (aligned to the reference *E. coli* str. K-12 genome), HotJaphi29 polymerase (red; from top to bottom: E1, E5, E26, E18, E22) and single cell amplified with Equiphi29 DNA polymerase (blue; from top to bottom: E14, E24, E32, E38, E45). **(B)** Amplification completeness. **(C)** Agarose gel analysis of the amplification efficiency of HotJa and Equiphi29 DNA polymerase; 16s PCR indicates the PCR results with primers 27F and 1492R, *uspA* PCR indicates the PCR results with primers uspA-F and uspA-R.

In this study, iSGA has the characteristics of high-temperature amplification capacity, low-cost and efficient MDA amplification capability. Firstly, the MDA amplification is routinely conducted at 30°C, which is the optimal temperature for the activity of phi29 DNA polymerase used (Alsmadi et al., 2009). We raised the amplification reaction temperature to 40°C and 50°C in *E. coli* MDA reactions (Figure 5) to eliminate or reduce nonspecific template-independent priming. We generated 5 iSGA amplified with HotJa Phi29 DNA polymerase at 40°C and 50°C, respectively. And 9 iSGA were amplified with HotJa Phi29 DNA polymerase at 30°C for control. Agarose gel electrophoresis of MDA and PCR products revealed that 5 out of 5 samples and 3 out of 5 samples generated typical MDA and PCR products bands at 40°C and 50°C amplification, respectively. For control, 6 out of 9 samples were amplified successfully at 30°C. Those results revealed that the amplification success rate at 40°C was the highest (about 100%) compared to that of 30°C (67%) and 50°C (60%) (Figure 5). Secondly, iSGA was low-cost, compared with EquiPhi29 DNA polymerase (the most efficient enzyme reported so far). Specifically, for EquiPhi29 DNA polymerase, each reaction to amplify sample DNA costs $4.79, while HotJa Phi29 DNA polymerase costs merely $0.44 (Table 3). Thirdly, iSGA has efficient MDA amplification capability. For *E. coli* samples, we generated 9 iSGA amplified with HotJa Phi29 DNA Polymerase and also 9 iSGA amplified with EquiPhi29 DNA polymerase for control. Agarose gel electrophoresis of MDA and PCR products revealed that, for both HotJa Phi29 DNA Polymerase group and control group, 9 out of 9 samples produced typical MDA and PCR product bands (Figure 6C). The marker gene fragments amplification was successful in samples with cells, but not in blank samples (including both those containing all reagents except sorted cells and those sorted cell-free droplet), and no contamination was detected in any of the samples based on sequencing of 16S rRNA amplification (Figure 6C). After the shotgun sequencing, no apparent contamination was detected in these reads, and average read coverage for the HotJa Phi29 DNA Polymerase group is much higher than the control group (95% versus 33%; *p* < 0.05; *t*-test) (Figures 6A, B; Table 3). Therefore, in comparison with EquiPhi29 DNA polymerase, the use of this enzyme provides better genome recovery for single bacterial benchmark strains.

**TABLE 3:** Comparing the Performance of iSGA and Equiphi29 DNA polymerase for single-cell genome amplification.

	iSGA	Equiphi29DNA polymerase
Enzyme cost	$0.44/reaction	$4.79/reaction
Amplification temperature	40°C	30°C
Coverage	90%	40%

**FIGURE 7:**
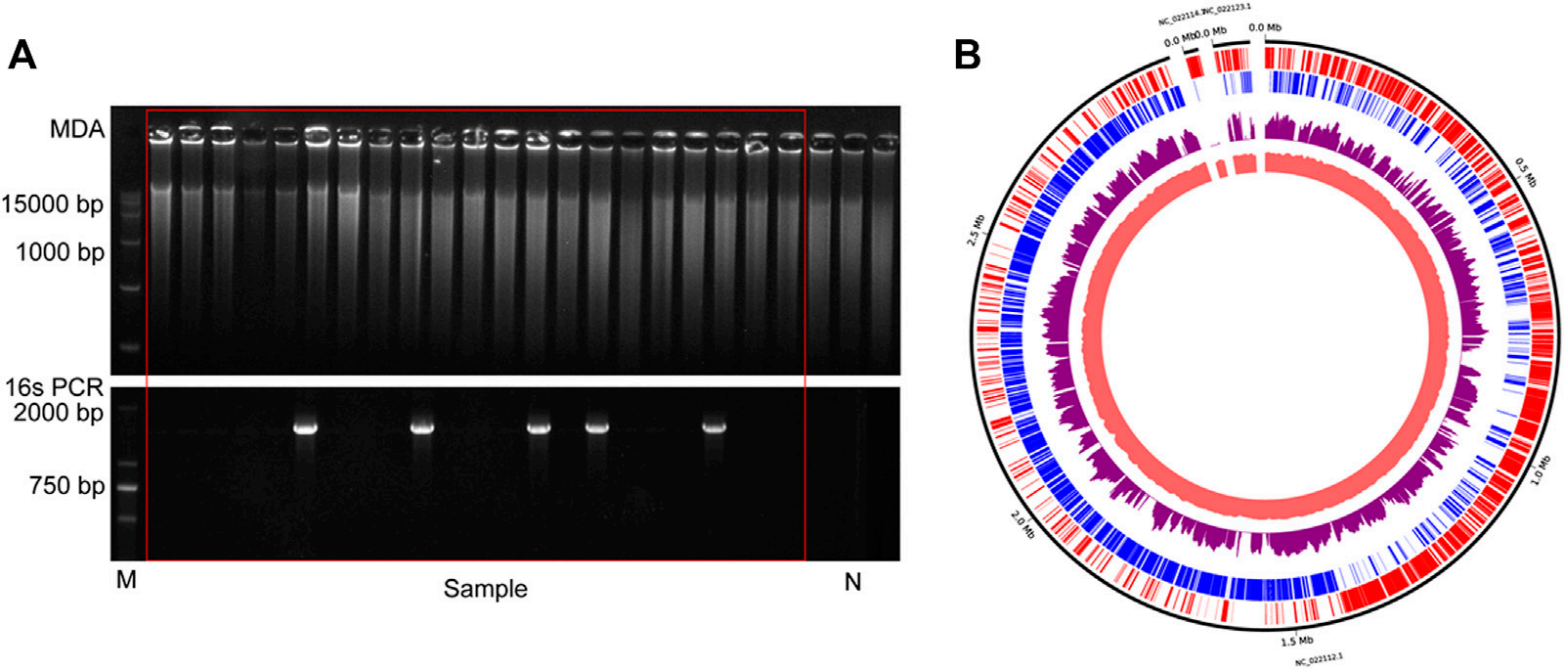
The results of commercial probiotic product. **(A)** Agarose gel analysis of the amplification efficiency of MDA and16S rRNA. N indicates the results of negative control. **(B)**The distribution of mapped sequencing reads on the *L. paracasei* genome, which was amplified with HotJa phi29 DNA polymerase (purple). The inner circles are the local GC content (Indian Red) and the outer two circles are genes on the forward strands (Red) and reverse strands (Blue).

To probe the value of the improved phi29 polymerase on commercial products, we chose a probiotic product (from EASTSEA PHARMA^®^) as an example. In this experiment, single probiotic cell was sorted followed by lysis and MDA. We generated 5 iSGA amplified with HotJa Phi29 DNA Polymerase. The assembled contigs revealed that these probiotic cells are Gram positive bacteria that mostly harbor low-GC% genomes (Table 4). And the completeness can reach 93.59% for the assembled genomes. We have shown here that the use of HotJa Phi29 DNA polymerase provides better genome recovery while maintaining similar fidelity for probiotic bacterial strains (Table 4; Figure 7).

**TABLE 4:** Precise one-cell genome sequencing of single bacterial cells directly from commercial probiotic products via iSGA.

Sample ID	Gram stain	Genus	GC%	Contamination	Coverage (%)
D6	positive	*L. paracasei*	45.89	8.52	93.59
D10	positive	*L. plantarum*	44.49	4.75	80.84
D14	positive	*L. rhamnosus*	46.34	1.94	87.44
D16	positive	*L. plantarum*	44.50	3.34	87.05
D20	positive	*L. paracasei*	46.27	3.20	89.91

## 4 Discussion

Here, we present a strategy to improve the phi29 DNA polymerase properties to enable its practical application in single cell amplification. We systematically explored several approaches, including (*i*) introduction of novel disulfide bonds into phi29 DNA polymerase by rational design, (*ii*) construction of phi29 DNA polymerase fused with the GB1 domain, and (*iii*) process engineering including efficient decontamination method and buffer modification.

The ability to improve “stability” and “durability” of an enzyme by computational modeling is of high value. Although disulfide bonds can improve protein stability (Perry and Wetzel, 1984; Matsumura et al., 1989; Liu et al., 2019), such as in the case of human neuroglobulins (Perry and Wetzel, 1984), and enhance the structural stability to improve the durability of enzyme activity (Zhang et al., 2020), it is not clear whether and how the engineered structure introduced by disulfide bonds is linked to the activity and amplification ability of enzymes. Our findings here suggest that the activity and amplification ability of enzymes can be greatly improved by constructing novel, *in silico* designed disulfide bonds, without necessarily sacrificing the level of protein over-expression. Notably, not all such disulfide bonds are created equal. For example, among the 12 new disulfide bonds tested, those formed in a region of primary sequence of phi29 DNA polymerase between residues 137 and 377 brought the most improvement in enzyme amplification ability. This reveals the important role of this domain in amplification ability of phi29 DNA polymerase activity. In addition, the introduced small GB1 domain was constructed that enhanced the stability and solubility of phi29 DNA polymerase and did not affect its activity, which is very effective for improving protein expression.

Preparation of phi29 DNA polymerase free of amplifiable DNA through UV irradiation was adopted for the reagents, including phi29 DNA polymerase, dNTPs, and primers (Wang et al., 2017). In this study, the removal of contaminating DNA from Phi29 DNA polymerase was achieved by combining UV irradiation. The procedure was conducted with the XL-1500 Spectrolinker Sanitizing Cabinet (Spectro-UV, New York, USA). This treatment significantly reduced amplifiable DNA in the polymerase without loss of activity by controlling exposure time and conditions. This suggests that UV irradiation through controlling exposure time and conditions could be easily applied in general to the DNA decontamination of polymerases and contamination from laboratory environments, tools and reagents.

The appropriate concentration of salt ions in the enzyme storage buffer plays a key role in the activity and stability of the enzyme (Gao et al., 2021). Salt stress caused by high salt concentration will affect the structural integrity of the polymerase, hindering the anchoring of DNA on the polymerase, and replication initiation (Pessôa Filho et al., 2011). While low salt concentrations may cause protein aggregation due to the salting-in effect (Xu et al., 2015). Thus, the amplification efficiency of phi29 DNA polymerase decreases with the increase of salt concentration (from 100 mM to 300 mM), because high salt concentration will disrupt the protein-DNA interaction and hamper polymerization.

Single-cell genomic whole genome amplification (WGA) is a challenging process and suffers from many significant limitations, such as low amplification efficiency, low amplification coverage, contamination, etc. In this study, iSGA was developed and showed significantly better coverage (99.75%) under higher temperature (40°C) through phi29 DNA polymerase engineering and process engineering. iSGA is more efficient and robust than the wild-type phi29 DNA polymerase, and it is 2.03-fold more efficient and 10.89-fold cheaper than ThermoFisher kit which is the most effective enzyme that has been reported. These advantages promise its broad applications in industrial single-cell sequencing.

## Data Availability

The datasets presented in this study can be found in online repositories. The names of the repository/repositories and accession number(s) can be found below: https://www.ncbi.nlm.nih.gov/, PRJNA961914 https://www.ncbi.nlm.nih.gov/, PRJNA961915.
