# Corrigendum: Improved single-cell genome amplification by a high-efficiency phi29 DNA polymerase

**DOI:** 10.3389/fbioe.2023.1263634

**Published:** 2023-08-28

**Authors:** Jia Zhang, Xiaolu Su, Yefei Wang, Xiaohang Wang, Shiqi Zhou, Hui Jia, Xiaoyan Jing, Yanhai Gong, Jichao Wang, Jian Xu

**Affiliations:** ^1^ Single-Cell Center, CAS Key Laboratory of Biofuels, Shandong Key Laboratory of Energy Genetics, Qingdao Institute of Bioenergy and Bioprocess Technology, Chinese Academy of Sciences, Qingdao, Shandong, China; ^2^ Shandong Energy Institute, Qingdao, Shandong, China; ^3^ Qingdao New Energy Shandong Laboratory, Qingdao, Shandong, China; ^4^ University of Chinese Academy of Sciences, Beijing, China; ^5^ CAS Key Laboratory of Biofuels and Shandong Key Laboratory of Synthetic Biology, Qingdao Institute of Bioenergy and Bioprocess Technology, Chinese Academy of Sciences, Qingdao, Shandong, China; ^6^ State Key Laboratory of Microbial Technology, Shandong University, Qingdao, Shandong, China

**Keywords:** single-cell genome amplification, phi29 DNA polymerase, disulfide bond, GB1 fusion protein, process engineering high-efficiency phi29 DNA polymerase

In the published article, Xiaolu Su and Yefei Wang were not listed as co-first authors. This has now been rectified.

In addition, there was an error in the **Funding** statement. National Natural Science Foundation of China (31872725 to YW) and Shandong Energy Institute (SEI) (SEI I202102 to YW) were not included. The correct Funding statement appears below:

This study was funded by National Key R&D Program Young Scientists Project of China (No. 2021YFD1900400 to XJ), Shandong Provincial Natural Science Foundation (ZR2021MC156 to JZ), National Natural Science Foundation of China (32030003 to JX and 32270109 to XJ), National Natural Science Foundation of China (31872725 to YW) and Shandong Energy Institute (SEI) (SEI I202102 to YW).

The authors apologize for this error and state that this does not change the scientific conclusions of the article in any way. The original article has been updated.

