# Supplementary material for: Improved single-cell genome amplification by a high-efficiency phi29 DNA polymerase
Source: Front Bioeng Biotechnol. 2023 Jun 29;11:1233856. doi: 10.3389/fbioe.2023.1233856 (PMC10347390; doi:10.3389/fbioe.2023.1233856)
Supplement: Supplementary file 1 [file DataSheet1.docx]

Supplementary Materials:

**The plasmids and strains constructed in this study**

Briefly, pET-21c(+)-phi29 wild type was constructed by ligation of the phi29 wild type fragment and the pET-21c(+) plasmid with the restriction sites of *Bam* HI and *Xho* I. Plasmids pZJphi29001 to pZJphi29021 were constructed for the expression of phi29 with corresponding mutations as shown in **Table S2**.

**The strains constructed in this study**

The above plasmids were transformed into *E. coli* BL21(DE3) by the heat shock method. The transformants were isolated on LB medium supplemented with ampicillin and validated by PCR. After the plasmids were transformed into *E. coli* BL21(DE3), corresponding strains were obtained as shown in **Table S3**.


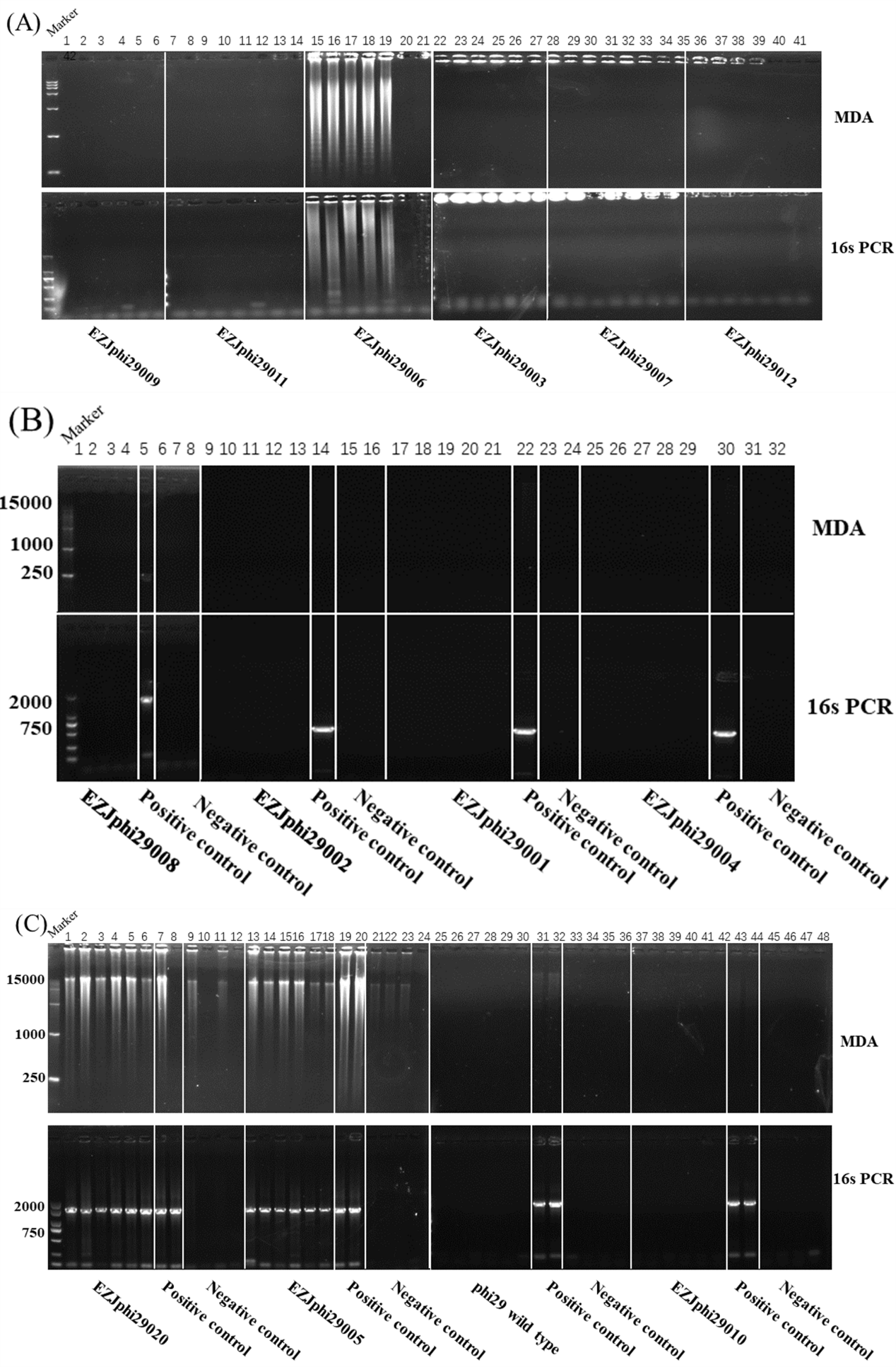


**Fig.S1 Agarose gel analysis of the amplification efficiency of various phi29 mutant variants.**

(A) Lane 1-6 for MDA reaction performed by EZJphi29009, lane 7-14 for MDA reaction performed by EZJphi29011, lane 15-21 for MDA reaction performed by EZJphi29006, lane 22-27 for MDA reaction performed by EZJphi29003, lane 28-35 for MDA reaction performed by EZJphi29007, lane 36-42 for MDA reaction performed by EZJphi29012, single cell was amplified by phi29 mutant variants. (B) Lane 1-8 for MDA reaction performed by EZJphi29008, lane 9-16 for MDA reaction performed by EZJphi29002, lane 17-24 for MDA reaction performed by EZJphi29001, lane 25-32 for MDA reaction performed by EZJphi29004. Lane 1-4, 9-13, 17-21 and 25-29, single cell was amplified by phi29 mutant variants. Lane 5, 14, 22 and 30 for positive control. Lane 6-8, 15-16, 23-24 and 31-32 for negative control. (C) Lane 1-12 for MDA reaction performed by EZJphi29020, lane 13-24 for MDA reaction performed by EZJphi29005, lane 25-36 for MDA reaction performed by wild type phi29, lane 37-48 for MDA reaction performed by EZJphi29010. Lane 1-6, 13-18, 25-30 and 37-42, single cell was amplified by phi29 mutant variants. Lane 7-8, 19-20, 31-32 and 43-44 for positive control. Lane 9-12, 21-24, 33-36 and 45-48 for negative control. 16s PCR indicates the PCR results with primers 27F and 1492R.


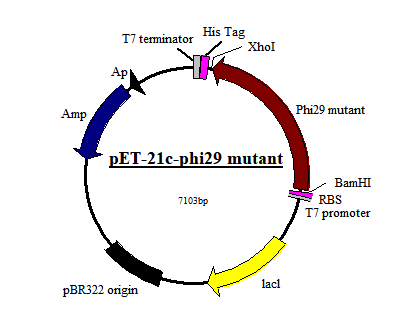


**Fig.S2 Schematic plasmid map for the major features present in phi29 mutant expression vector.**

**Table S1.** **The primers used in this study.**

| **Primer names** | **Primer sequences** |
| --- | --- |
| K7C-A56C-F1 | ACACATGCCGCGTTGCATGTACTCTTGCGA |
| K7C-A56C-R1 | TCGCAAGAGTACATGCAACGCGGCATGTGT |
| K7C-A56C-F2 | CTGAAAGTTCAGTGCGACCTGTACTTCCAC |
| K7C-A56C-R2 | GTGGAAGTACAGGTCGCACTGAACTTTCAG |
| V24C-L44C-F1 | TGGAAGATTGCCGTTGCTGGGCGTACGGCT |
| V24C-L44C-R1 | AGCCGTACGCCCAGCAACGGCAATCTTCCA |
| V24C-L44C-F2 | AAATCGGCAACAGCTGCGACGAATTCATGG |
| V24C-L44C-R2 | CCATGAATTCGTCGCAGCTGTTGCCGATTT |
| Y59C-S122C-F1 | TCAGGCGGACCTGTGCTTCCACAACCTGAA |
| Y59C-S122C-R1 | TTCAGGTTGTGGAAGCACAGGTCCGCCTGA |
| Y59C-S122C-F2 | ACCGTTATCTACGACTGCCTGAAAAAACTG |
| Y59C-S122C-R2 | CAGTTTTTTCAGGCAGTCGTAGATAACGGT |
| E75C-L406C-F1 | ATCATCAACTGGCTGTGCCGTAACGGCTTT |
| E75C-L406C-R1 | AAAGCCGTTACGGCACAGCCAGTTGATGAT |
| E75C-L406C-F2 | CAAAGTTCCGTACTGCAAAGAAAACGGCGC |
| E75C-L406C-R2 | GCGCCGTTTTCTTTGCAGTACGGAACTTTG |
| F137C-A377C-F1 | AGATCGCGAAAGATTGCAAACTGACCGTTC |
| F137C-A377C-R1 | GAACGGTCAGTTTGCAATCTTTCGCGATCT |
| F137C-A377C-F2 | CACTAGCGAAGGTTGCATTAAACAGCTGGC |
| F137C-A377C-R2 | GCCAGCTGTTTAATGCAACCTTCGCTAGTG |
| S194C-S388C-F1 | CGCGGGCAGCGATTGCCTGAAAGGCTTCAA |
| S194C-S388C-R1 | TTGAAGCCTTTCAGGCAATCGCTGCCCGCG |
| S194C-S388C-F2 | AAACTGATGCTGAACTGCCTGTACGGTAAA |
| S194C-S388C-R2 | TTTACCGTACAGGCAGTTCAGCATCAGTTT |
| L253C-D458C-F1 | TTCGACGTGAACTCCTGCTACCCGGCGCAG |
| L253C-D458C-R1 | CTGCGCCGGGTAGCAGGAGTTCACGTCGAA |
| L253C-D458C-F2 | CTACTGCGATACCTGCTCTATCCACCTGAC |
| L253C-D458C-R2 | GTCAGGTGGATAGAGCAGGTATCGCAGTAG |
| Y254C-Y390C-F1 | GACGTGAACTCCCTGTGCCCGGCGCAGATG |
| Y254C-Y390C-R1 | CATCTGCGCCGGGCACAGGGAGTTCACGTC |
| Y254C-Y390C-F2 | GCTGAACTCCCTGTGCGGTAAATTTGCTTC |
| Y254C-Y390C-R2 | GAAGCAAATTTACCGCACAGGGAGTTCAGC |
| M258C-F360C-F1 | TACCCGGCGCAGTGCTACTCTCGTCTGCTG |
| M258C-F360C-R1 | CAGCAGACGAGAGTAGCACTGCGCCGGGTA |
| M258C-F360C-F2 | GCCACCACGGGCCTGTGCAAAGACTTCATC |
| M258C-F360C-R2 | GATGAAGTCTTTGCACAGGCCCGTGGTGGC |
| Y281C-K352C-F1 | TTTGGGACGAAGACTGCCCACTGCACATCC |
| Y281C-K352C-R1 | GGATGTGCAGTGGGCAGTCTTCGTCCCAAA |
| Y281C-K352C-F2 | CATCTCCGGCCTGTGCTTCAAAGCCACCAC |
| Y281C-K352C-R2 | GTGGTGGCTTTGAAGCACAGGCCGGAGATG |
| Y298C-H339C-F1 | AACTGAAAGAGGGTTGCATCCCGACCATCC |
| Y298C-H339C-R1 | GGATGGTCGGGATGCAACCCTCTTTCAGTT |
| Y298C-H339C-F2 | CTGATGAAAGAATGCTATGACCTGTACAAC |
| Y298C-H339C-R2 | GTTGTACAGGTCATAGCATTCTTTCATCAG |
| V331C-A435C-F1 | TGGCTGTCCAACTGCGACCTGGAACTGATG |
| V331C-A435C-R1 | CATCAGTTCCAGGTCGCAGTTGGACAGCCA |
| V331C-A435C-F2 | CGTTTTCATCACCTGCTGGGCTCGTTACAC |
| V331C-A435C-R2 | GTGTAACGAGCCCAGCAGGTGATGAAAACG |
| M8R-F | CACATGCCGCGTAAACGTTACTCTTGCGATTTCG |
| M8R-R | CGAAATCGCAAGAGTAACGTTTACGCGGCATGTG |
| V51A-F | ATTCATGGCCTGGGCGCTGAAAGTTCAGGCG |
| V51A-R | CGCCTGAACTTTCAGCGCCCAGGCCATGAAT |
| M97T-F | ATCATTTCCCGCACCGGTCAGTGGTATATG |
| M97T-R | CATATACCACTGACCGGTGCGGGAAATGAT |
| G197D-F | AGCGATAGCCTGAAAGACTTCAAAGACATCATC |
| G197D-R | GATGATGTCTTTGAAGTCTTTCAGGCTATCGCT |
| E221K-F | TGGGTCTGGACAAAAAAGTGCGCTATGCGT |
| E221K-R | ACGCATAGCGCACTTTTTTGTCCAGACCCA |
| Q497P-F | AAATATCTGCGTCCGAAAACCTACATCC |
| Q497P-R | GGATGTAGGTTTTCGGACGCAGATATTT |
| K512E-F | AGAAGTTGATGGCGAACTGGTGGAAGGTAG |
| K512E-R | CTACCTTCCACCAGTTCGCCATCAACTTCT |
| F526L-F | ACACCGACATCAAACTGAGCGTGAAATGCG |
| F526L-R | CGCATTTCACGCTCAGTTTGATGTCGGTGT |
| 036A-F | GCTGCTGCCCATTCGGATCCGACCCAT |
| 036A-R | GGAGGCAGCAAACACATGCCGCGTAAAC |
| 036A-1 | GCATGTGTTTGCTGCCTCCGCCACCCGA |
| 036A-2 | GCGGCGGTAGTGGCGGTGGCGGTTCGGGCGGCGGTGGTTCGGGTGGCGGAGGCAGC |
| 036A-3 | CACCGCCACTACCGCCGCCACCGCTGCCACCGCCTCCGCTCCCACCACCTTCTGTGACAGTGAAGGTTTTGGTTG |
| 036A-4 | GTATGCAAACGACAACGGTGTTGATGGAGAATGGACATACGATGACGCAACCAAAACCTTCACTGTCACAGAA |
| 036A-5 | TCAACACCGTTGTCGTTTGCATACTGTTTAAATACTTTTTCAGCGGTAGCAGCGTCAACAGCTTCTGTTGTTG |
| 036A-6 | GTTCAGGCGGTAGTTATAAATTGATCCTGAACGGCAAAACTTTGAAGGGCGAAACAACAACAGAAGCTGTTGACGCTG |
| 036A-7 | AGGATCAATTTATAACTACCGCCTGAACCACCGGATCCGCCCACGGCATGATGGTGGTGGTGATGATGATGATG |
| 036A-8 | TCCGAATGGGCAGCAGCCATCATCATCATCATCACCACCAC |
| 27F | AGAGTTTGATCCTGGCTCAG |
| 1492R | TACGGYTACCTTGTTACGACTT |
| uspA-F | CCGATACGCTGCCAATCAGT |
| uspA-R | ACGCAGACCGTAGGCCAGAT |

**Table S2: Plasmids used in this study**

| Plasmids | Selection marker and description | Reference |
| --- | --- | --- |
| pET-21c(+)-phi29 wild type | *AmpR*, phi29 | This study |
| pZJphi29001 | *AmpR*, phi29-K7C-A56C | This study |
| pZJphi29002 | *AmpR*, phi29-V24C-L44C | This study |
| pZJphi29003 | *AmpR*, phi29-Y59C-S122C | This study |
| pZJphi29004 | *AmpR*, phi29-E75C-L406C | This study |
| pZJphi29005 | *AmpR*, phi29-F137C-A377C | This study |
| pZJphi29006 | *AmpR*, phi29-S194C-S388C | This study |
| pZJphi29007 | *AmpR*, phi29-L253C-D458C | This study |
| pZJphi29008 | *AmpR*, phi29-Y254C-Y390C | This study |
| pZJphi29009 | *AmpR*, phi29-M258C-F360C | This study |
| pZJphi29010 | *AmpR*, phi29-Y281C-K352C | This study |
| pZJphi29011 | *AmpR*, phi29-Y298C-H339C | This study |
| pZJphi29012 | *AmpR*, phi29-V331C-A435C | This study |
| pZJphi29013 | *AmpR*, phi29-F137C-A377C-M8R | This study |
| pZJphi29014 | *AmpR*, phi29-F137C-A377C-M8R-V51A | This study |
| pZJphi29015 | *AmpR*, phi29-F137C-A377C-M8R-V51A-M97T | This study |
| pZJphi29016 | *AmpR*, phi29-F137C-A377C-M8R-V51A-M97T-G197D | This study |
| pZJphi29017 | *AmpR*, phi29-F137C-A377C-M8R-V51A-M97T-G197D-E221K | This study |
| pZJphi29018 | *AmpR*, phi29-F137C-A377C-M8R-V51A-M97T-G197D-E221K-Q497P | This study |
| pZJphi29019 | *AmpR*, phi29-F137C-A377C-M8R-V51A-M97T-G197D-E221K-Q497P-K512E | This study |
| pZJphi29020 | *AmpR*, phi29-F137C-A377C-M8R-V51A-M97T-G197D-E221K-Q497P-K512E-F526L | This study |
| pZJphi29021 | *AmpR*, phi29-F137C-A377C-M8R-V51A-M97T-G197D-E221K-Q497P-K512E-F526L-GB1 | This study |

**Table S3. The strains constructed in this study.**

| **Strains** | **Selection marker and plasmids** | **Competent cell** |
| --- | --- | --- |
| phi29 wild type | AmpR, pET-21c(+)-phi29 wild type | *E. coli* BL21(DE3) |
| EZJphi29001 | AmpR, pZJphi29001 | *E. coli* BL21(DE3) |
| EZJphi29002 | AmpR, pZJphi29002 | *E. coli* BL21(DE3) |
| EZJphi29003 | AmpR, pZJphi29003 | *E. coli* BL21(DE3) |
| EZJphi29004 | AmpR, pZJphi29004 | *E. coli* BL21(DE3) |
| EZJphi29005 | AmpR, pZJphi29005 | *E. coli* BL21(DE3) |
| EZJphi29006 | AmpR, pZJphi29006 | *E. coli* BL21(DE3) |
| EZJphi29007 | AmpR, pZJphi29007 | *E. coli* BL21(DE3) |
| EZJphi29008 | AmpR, pZJphi29008 | *E. coli* BL21(DE3) |
| EZJphi29009 | AmpR, pZJphi29009 | *E. coli* BL21(DE3) |
| EZJphi29010 | AmpR, pZJphi29010 | *E. coli* BL21(DE3) |
| EZJphi29011 | AmpR, pZJphi29011 | *E. coli* BL21(DE3) |
| EZJphi29012 | AmpR, pZJphi29012 | *E. coli* BL21(DE3) |
| EZJphi29020 | AmpR, pZJphi29020 | *E. coli* BL21(DE3) |
| EZJphi29021 | AmpR, pZJphi29021 | *E. coli* BL21(DE3) |
